# Human Mesenchymal Stem Cells Growth and Osteogenic Differentiation on Piezoelectric Poly(vinylidene fluoride) Microsphere Substrates

**DOI:** 10.3390/ijms18112391

**Published:** 2017-11-11

**Authors:** R. Sobreiro-Almeida, M. N. Tamaño-Machiavello, E. O. Carvalho, L. Cordón, S. Doria, L. Senent, D. M. Correia, C. Ribeiro, S. Lanceros-Méndez, R. Sabater i Serra, J. L. Gomez Ribelles, A. Sempere

**Affiliations:** 1 Centro/Departamento de Física, Universidade do Minho, 4710-057 Braga, Portugal; rita.isaias.almeida@hotmail.com (R.S.-A.); estela.carvalhoo@hotmail.com (E.O.C.); d.correia@fisica.uminho.pt (D.M.C.); 2 Centre for Biomaterials and Tissue Engineering, CBIT, Universitat Politècnica de València, 46022 Valencia, Spain; noeltm@gmail.com (M.N.T.-M.); sergidobe@msn.com (S.D.); rsabater@die.upv.es (R.S.iS.); jlgomez@ter.upv.es (J.L.G.R.); 3 Hematology Research Group, Instituto de Investigación Sanitaria La Fe, 46026 Valencia, Spain; lou.cordon@gmail.com (L.C.); senent_leo@gva.es (L.S.); sempere_amp@gva.es (A.S.); 4 Centro de Investigación Biomédica en Red de Cáncer (CIBERONC), Instituto Carlos III, 28029 Madrid, Spain; 5 Hematology Department, Hospital Universitario y Politécnico La Fe, 46026 Valencia, Spain; 6 Centro/Departamento de Química, Universidade do Minho, Campus de Gualtar, 4710-057 Braga, Portugal; 7 CEB—Centre of Biological Engineering, University of Minho, Campus de Gualtar, 4710-057 Braga, Portugal; 8 BCMaterials, Parque Científico y Tecnológico de Bizkaia, 48160 Derio, Spain; lanceros@fisica.uminho.pt; 9 IKERBASQUE, Basque Foundation for Science, 48013 Bilbao, Spain; 10 Biomedical Research Networking Center on Bioengineering, Biomaterials and Nanomedicine (CIBER-BBN), 46022 Valencia, Spain

**Keywords:** tissue engineering, bone differentiation, poly(vinylidene fluoride), microspheres

## Abstract

The aim of this work was to determine the influence of the biomaterial environment on human mesenchymal stem cell (hMSC) fate when cultured in supports with varying topography. Poly(vinylidene fluoride) (PVDF) culture supports were prepared with structures ranging between 2D and 3D, based on PVDF films on which PVDF microspheres were deposited with varying surface density. Maintenance of multipotentiality when cultured in expansion medium was studied by flow cytometry monitoring the expression of characteristic hMSCs markers, and revealed that cells were losing their characteristic surface markers on these supports. Cell morphology was assessed by scanning electron microscopy (SEM). Alkaline phosphatase activity was also assessed after seven days of culture on expansion medium. On the other hand, osteoblastic differentiation was monitored while culturing in osteogenic medium after cells reached confluence. Osteocalcin immunocytochemistry and alizarin red assays were performed. We show that flow cytometry is a suitable technique for the study of the differentiation of hMSC seeded onto biomaterials, giving a quantitative reliable analysis of hMSC-associated markers. We also show that electrosprayed piezoelectric poly(vinylidene fluoride) is a suitable support for tissue engineering purposes, as hMSCs can proliferate, be viable and undergo osteogenic differentiation when chemically stimulated.

## 1. Introduction

Tissue engineering combines specially designed biomaterials with cells to augment, replace or reconstruct damaged or diseased tissues. Human Mesenchymal Stem Cells (hMSCs) have attracted strong interest in the scientific community in the past few years due to their differentiation potential towards cells belonging to musculoskeletal lineages, such as osteoblasts, adipocytes and chondrocytes [1,2]. In order to induce hMSC differentiation to the desired lineage, extracellular stimuli are required, including chemical and physical cues. In addition, biomaterial properties have been demonstrated to modulate short-term cell functions, and in the last years they have evolved from basic supporting materials to biofunctional materials [3]. It has been extensively proven that hMSCs can be expanded in adherent culture flasks to high cell numbers while maintaining their multipotentiality, i.e., their ability to differentiate to osteoblasts, adipocytes and chondrocytes in specific differentiation media [4]. Nevertheless, the range of biomaterials used as supports in cell culture and as a vehicle for cell transplant to the organism in tissue engineering therapies are very broad, including many different chemical structures, surface topographies and 3D arrangements such as gels or scaffolds. During culture, the influence of cell–material interaction on the fate of hMSCs is not well understood. In particular, if cell culture into 3D supports compromises the cells towards one of the particular lineages [5].

Synthetic polymers have been increasingly used as biomaterials to serve as a supports for cell growth and differentiation due to their intrinsic attractive properties compared to inorganic materials; they can be manufactured into complex shapes and their physicochemical properties can easily be tailored [6]; as well as for their surface properties. It has been demonstrated that biomaterial design and surface characteristics (including roughness, surface charge or chemistry and wettability) can modulate the cell function [7,8]. In particular, biomaterial surface properties are linked to cell adhesion, morphology, proliferation and differentiation [9].

Furthermore, new structures are being produced in order to offer adequate cell supports which could mimic their natural niche in a more realistic way. In particular, polymer microspheres have been increasingly used as supports for cell expansion and differentiation, holding and populating a higher number of cells than the traditional 3D scaffolds [10,11].

Furthermore, the intrinsic properties of specific polymers (such as smart polymers) can be taken to advantage of in order to influence the behavior and differentiation of cells. In particular, it has been demonstrated that the use of piezoelectric polymers as substrates/scaffolds enhances cell response since they have the ability to vary their surface charge when a mechanical load is applied, without the need of a power source or wires [12,13]. This electrical response can also be induced in related magnetic composites through the application of a magnetic field [14], allowing the tuning of cell response. Among these polymers, poly(vinylidene fluoride) (PVDF) is the one with the largest piezo-, pyro- and ferroelectric responses [15]. Also, this biocompatible polymer shows good osteoinductive results, not only on dynamic, but also on static cultures, proving that it is suitable for stem cell culture and differentiation into the osteogenic lineage [16,17,18,19,20].

However, it is important to have a precise and defined method to assess hMSC differentiation when cultured on diverse biomaterials. There are various methods for the identification, verification and characterization of hMSCs, such as immunofluorescence/immunocytochemistry, Western blot, protein arrays and real-time polymerase chain reaction (RT-PCR) [21,22,23]. Cytometry has emerged as a simple and more precise method to identify and evaluate hMSC differentiation as they change the expression of their classical surface antigens—CD105, CD73 and CD90—after culture on the biomaterial [24,25].

In this work, PVDF substrates have been produced that are somewhere in between 2D and 3D configurations; the cells can grow on flat surface and 3D spheres. In this way, the cells do not find just a flat surface to attach to, but do not find a typical 3D scaffold with attachments points all around the cells, either. They consist of a flat PVDF film on which PVDF microspheres have been randomly distributed with varying surface density. It is important to mention that with lower quantity of microspheres deposited on the film, the structures are closer to 2D, while structures with a higher quantity of microspheres deposited on the films are closer to 3D. Furthermore, by keeping the processing parameters constant, including deposition time, structures with the same characteristics area always obtained. The objective is to show if an increasing three-dimensional interactions between cells and biomaterial surfaces influence cell fate either in expansion or in an osteoblastic culture medium.

## 2. Results

### 2.1. PVDF Samples Characterization

PVDF microspheres have been produced by electrospray from a 7% *w*/*v* solution in *N*,*N*-dimethylformamide (DMF) and obtained according to [26] (Figure S1), with diameters between 0.81 ± 0.34 and 5.55 ± 2.34 μm, and an average diameter of 3.04 ± 1.70 μm.

The β-PVDF electroactive phase has shown superior results in MSC and MC3T3-E1 preosteoblasts cell culturing [12,18]. Thus, Fourier transformed infrared (FTIR) measurements were carried out in order to confirm the presence of this phase in the microspheres. The characteristic FTIR bands of PVDF provide an accurate confirmation that the β-phase was obtained [15]. The relative fraction of the β-phase of the microspheres was determined as explained in [26], showing an electroactive phase content around 70%.

Complementary to FTIR measurements, differential scanning calorimetry (DSC) was performed in order to identify and quantify the crystalline phase of PVDF microspheres following a procedure similar to the one in [26] (data not shown). The obtained degree of crystallinity for the electrosprayed microspheres is around 52%.

### 2.2. Characterization of the Mesenchymal Cells

The mesenchymal origin of the hMSCs was confirmed by the adherence of the cells to tissue culture polystyrene plates (TCPS), and their surface marker expression was analyzed by multiparametric flow cytometry (MFC).

**Figure 1:**
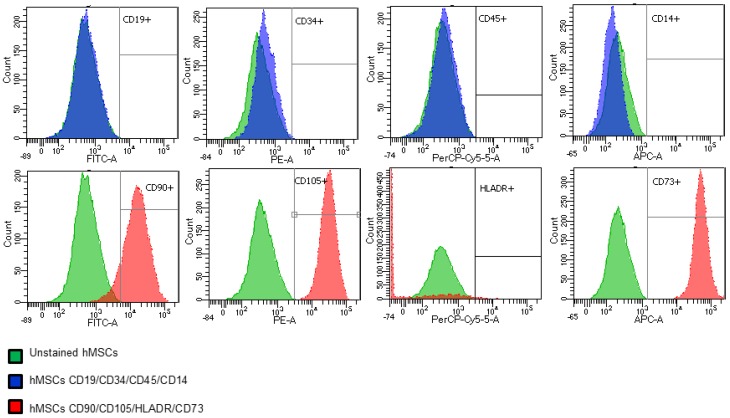
Flow cytometry histograms of merged samples of the hMSCs (passage 4) at day 0 of cell culture. Unstained hMSCs (**green**); hMSCs stained with CD19/CD34/CD45/CD14 (**blue**); hMSCs stained with CD90/CD105/HLADR/CD73 (**red**). Fluorescein isothiocyanate (FITC): CD19 and CD90; phycoerythrin (PE): CD34 and CD105; peridinin chlorophyll protein-cyanine5.5 (PerCP-Cy5.5): CD45 and HLA-DR; allophycocyanin (APC): CD14 and CD73.

The hMSCs are characterized by the expression of the monoclonal antibodies CD90, CD105 and CD73, and the lack of expression of CD14, CD19, CD34, CD45 and HLADR as proposed by The International Society of Cellular Therapy (ISCT) [24]. We show in Figure 1 MFC analysis of the hMSCs, performed after culture in TCPS at passage 4 when merging the data from the unstained and stained cells with the negative or the positive markers employing FACSDiva version 8 software (Becton Dickinson, San Jose, CA, USA). The histograms show the percentage of each antigen fluorochrome conjugated expressed by the hMSCs. The higher the value the higher the expression of cell antigens.

The percentage of expression of cell surface proteins (TCPS, passage 4) is shown in Table 1. Unstained hMSCs were employed as negative controls, showing no expression for the indicated markers. Stained hMSCs were positive for CD90, CD105 and CD73 whereas they were negative for hematopoietic markers. Notably, CD90 expression was lower than expected.

### 2.3. hMSCs Culture in Expansion Medium

#### 2.3.1. Cell Morphology

**Figure 2:**
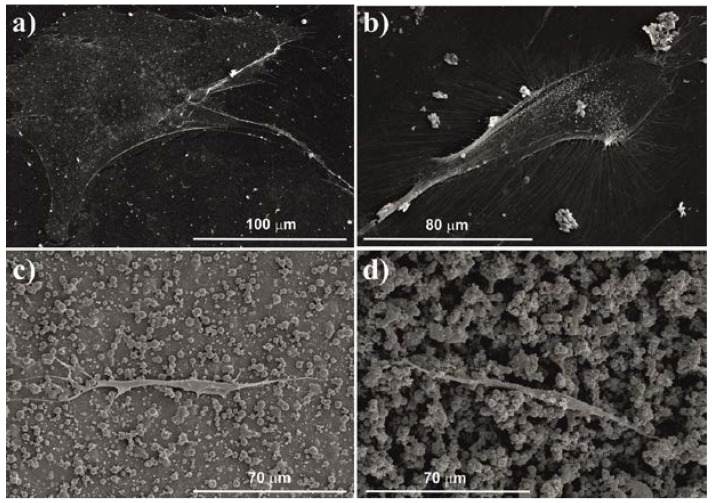
Overall cell morphology of hMSCs analyzed by SEM after four days of cell culture on: (**a**) glass; (**b**) β-PVDF film; (**c**) film with low density of PVDF microspheres; (**d**) film with high density of PVDF microspheres.

Overall cell morphology of the hMSCs seeded on the PVDF samples was visualized after four days of cell culture by scanning electron microscopy (SEM) (Figure 2). SEM images revealed that cells cultured on the microsphere films seem to elongate their adhesion points in order to find a suitable place to hold on to. In the film with high density of microspheres (HD-M), the cells became thinner and their bodies became less flattened and more elongated, compared to the film with low density of microspheres (LD-M). Figure 2c also shows that the cells were able to attach with their elongated filopodia while showing adhesion within the cell body to the film and microspheres. As there is no visible film in Figure 2d, the film with HD-M resembles a 3D environment, where the cells can only attach to the agglomerates of microspheres, and because of that, hMSCs adopt a particular shape. Contrarily, it is possible to verify that cells cultured in flat substrates (glass and β-PVDF film) showed a more spread and flattened morphology (Figure 2a,b respectively).

#### 2.3.2. Viability

**Figure 3:**
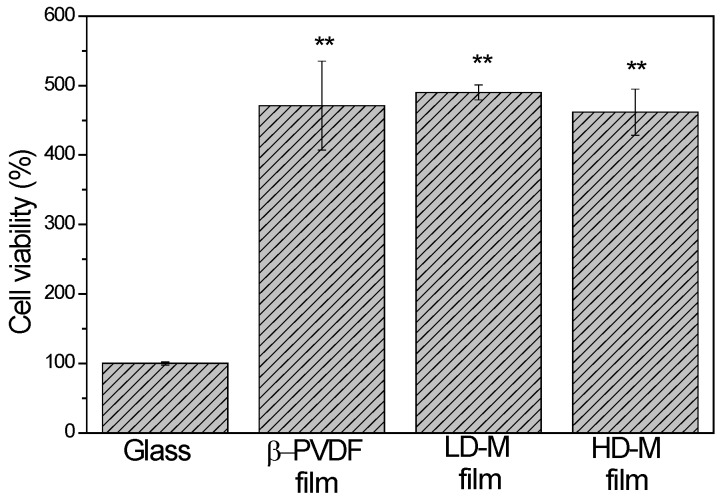
Cell viability for cells seeded on the PVDF samples and on glass covers (positive control) after four days of cell culture. Results are expressed as mean ± standard deviation with *n* = 3. ** *p* ≤ 0.01 vs. glass.

The viability of the attached cells on PVDF samples after four days of culture was also studied by 3-(4,5-dimethylthiazol-2-yl)-5-(3-carboxymethoxyphenyl)-2-(4-sulfophenyl)-2H-tetrazolium (MTS) assays (Figure 3). The results showed that PVDF is a suitable biomaterial for hMSC growth and survival, since the PVDF samples have an increase of ≅400% in the measured absorbance compared to cells seeded on glass covers. Furthermore, there are no significant statistical differences among the PVDF samples. This result corroborates other cell studies performed with flat PVDF substrates [12,26,27].

#### 2.3.3. MFC Analysis

**Figure 4:**
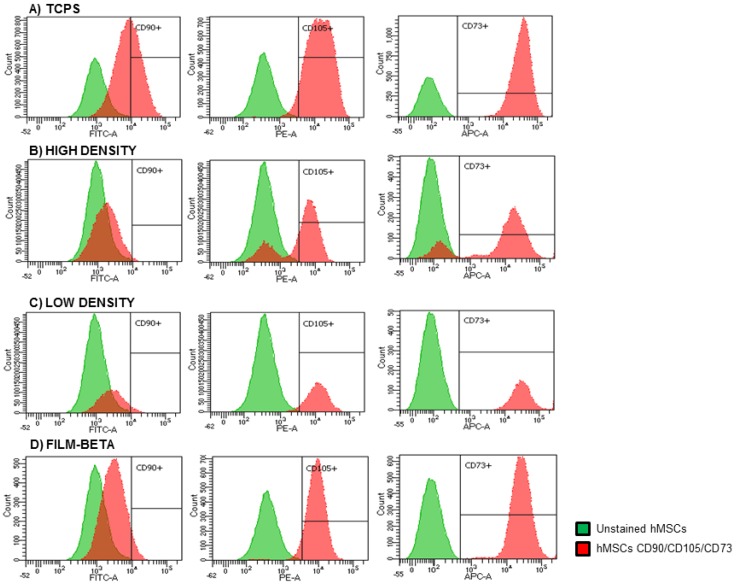
Flow cytometry histograms of merged samples of the unstained and stained hMSCs cultured at day 4 on (**a**) tissue culture polystyrene (TCPS); (**b**) low density of microparticles (LD-M) film; (**c**) high density of microparticles (HD-M) film; and (**d**) film-beta. Fluorescein isothiocyanate (FITC): CD90; phycoerythrin (PE): CD105; allophycocyanin (APC): CD73.

In order to evaluate the loss or maintenance of the hMSC antigens after four days of cell culture on TCPS and PVDF samples, cells were submitted to MFC analysis (Figure 4).

To assess whether films with adsorbed microparticles induce changes in cell marker expression, hMSCs were cultured for four days on different biomaterials: TCPS, low density, high density and film-beta were compared to stained cell control at day 0 (Figure 4).

The percentage of expression of CD90, CD105 and CD73 antigens in hMSCs is shown in Table 2. We observed that CD90 expression decreased significantly in hMSCs cultured on all samples, being more significantly diminished in HD-M, LD-M and film-beta with respect to the control at day 0. CD105 expression was lower in HD-M samples compared to the control at day 0. In the rest of the samples the decrease of this expression was inferior. HD-M sample showed less expression of CD73 compared with the other samples, which maintained similar levels to control at day 0.

#### 2.3.4. Alkaline Phosphatase Activity

**Figure 5:**
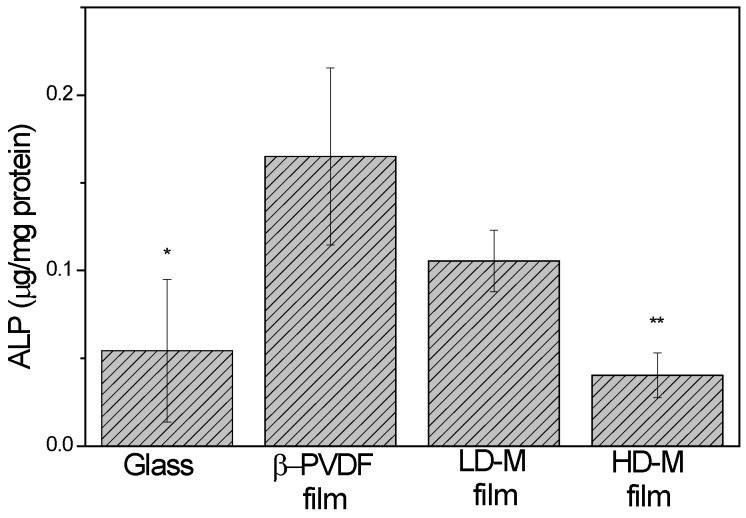
Alkaline phosphatase (ALP) activity in hMSCs cultured on different substrates for seven days. * The ALP activity of cells on β-PVDF film was significantly higher than that on high density (HD-M) film and glass (*p* < 0.05). Data are expressed as the mean standard deviation with *n* = 3. * *p* ≤ 0.05 vs. β-PVDF film and ** *p* ≤ 0.01 vs. β-PVDF film.

Alkaline phosphatase (ALP) is an enzyme that increases the local concentration of inorganic phosphatase and has been shown to be important in hard tissue formation and mineralization [28]. Furthermore, ALP activity is higher in mature osteoblastic cells than in preosteoblastic and mesenchymal cells [29]. Figure 5 shows ALP activity in hMSCs. The ALP activity on β-PVDF films was significantly higher than that on HD-M film and on glass (*p* < 0.05), but there was no significant difference among the other samples.

### 2.4. hMSCs Culture in Differentiation Medium

Expansion medium was changed by osteogenic medium when cells become confluent. Cell culture that continued with expansion medium for the same number of days was used as control.

#### 2.4.1. Osteocalcin Expression

**Figure 6:**
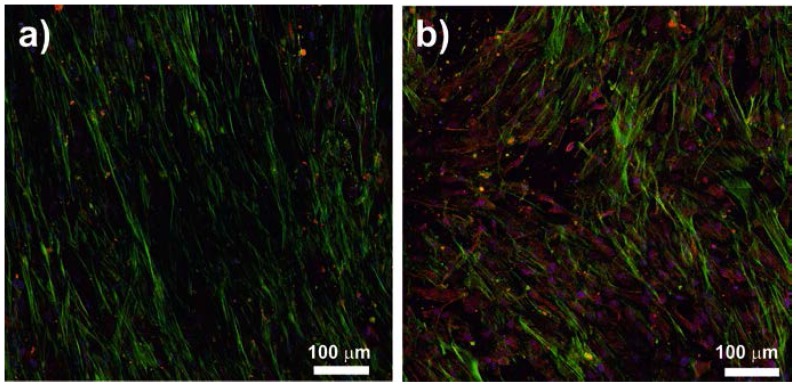
Confocal fluorescence microscopy images of cells after 14 days of culture (with differentiation medium) in: (**a**) glass; (**b**) high density of microparticles (HD-M) film. The picture shows bone-specific osteocalcin (red), the cells actin cytoskeleton (green) and the nucleus (blue). The scale bar (100 μm) is valid for all images.

Osteocalcin (OC) is a major bone protein and has an important function in the metabolism of mineralized tissues [30]. Therefore, to corroborate the results obtained by MFC analysis, after 14 days of osteogenic medium addition, an immunocytochemistry localization of osteocalcin was performed. The staining in β-phase PVDF film or in the two PVDF microsphere samples are similar to each other (Figure 6b shows the case of the (HD-M support) which is clearer than in the glass control (Figure 6a). The elongated morphology after osteogenic induction can also be observed through actin green staining. It is noteworthy that cells seeded on glass showed a much more organized morphology, when compared to those cultured on PVDF samples, which seem to swirl. Also, in the HD-M film this effect seemed to be enhanced, probably due to the fact that the cells are forced to elongate and grow depending on the relative placement of the microspheres. Given that these films have higher amounts of microspheres, cells do not have a flat surface to hold on and spread freely.

#### 2.4.2. Quantitative Analysis of Alizarin Red Staining

**Figure 7:**
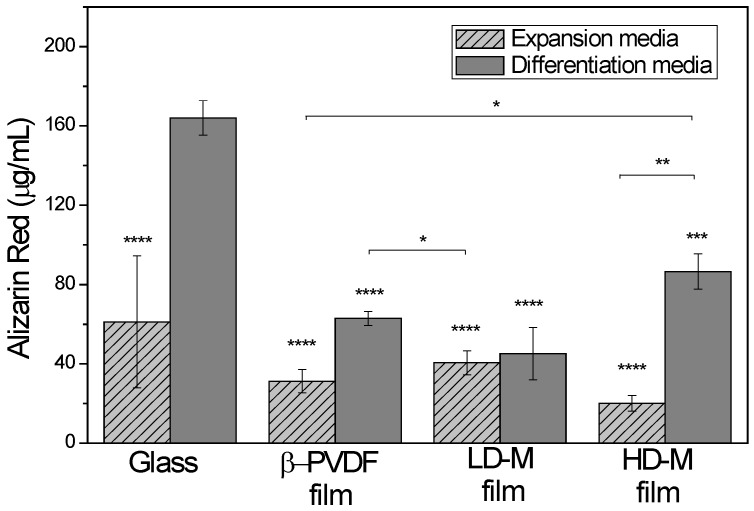
Alizarin red staining (ARS) acid extraction in hMSCs cultured in different substrates with osteogenic supplements (differentiation media) and without them (expansion media) after 14 days. Data are expressed as the mean ± standard deviation with *n* = 3. * *p* ≤ 0.05, ** *p* < 0.01, *** *p* ≤ 0.001 vs. Glass with differentiation media and **** *p* ≤ 0.0001 vs. glass with differentiation media.

Figure 7 shows the alizarin red staining (ARS) acid extraction in hMSCs cultured in different substrates with osteogenic supplements (differentiation medium) and without them (expansion medium) after 14 days. The mineralization was detected in all materials and in both media. Comparing the PVDF samples, higher ARS concentration was found in the HD-M samples in differentiation medium.

## 3. Discussion

In this work, the effect of PVDF’s surface topography for tissue engineering purposes has been studied. New quasi-3D substrates of microspheres were produced and hMSC osteogenic differentiation was induced in order to observe the potential of each biomaterial.

The morphology adopted by the cells on different materials has already proven to be fundamental for cell proliferation and fate [31]. The images have shown that different PVDF topographies were successfully obtained and that cells adopt different morphologies regarding the substrate they are cultured on (Figure 2). It has been demonstrated previously that disordered structures promote hMSCs to undergo osteogenic differentiation and that mechanotransductive events between the cell and the biomaterial are a key factor influencing cell fate [32]. In this context, it has been reported that increased contractility of hMSCs leads preferentially to osteogenesis, while low contractility leads to adipogenesis [33]. Throughout the years, a series of studies have been performed demonstrating that cell culture conditions that increased cytoskeletal tension promote osteogenesis; other studies have linked cytoskeletal tension to cell spreading. For instance, previous studies related matrix elasticity to cell differentiation showing that stiffer matrices increase cytoskeleton tension, thereby promoting osteogenesis, while softer matrices lead hMSCs to differentiate towards alternative lineages [34]. Furthermore, it has been proven that changes in cell shape can alone influence hMSC commitment between osteogenic and adipogenic differentiation [35]. Although the assessment of cell morphology in this study was done with few days of cell culture, it has been shown that cells cultured on flat supports already have a more spread and flattened morphology than cells seeded on microsphere substrates. Although the disordered nature of the microspheres do not induce any preferential orientation of the cells, those that are grown on these substrates in a similar way find a place to elongate their filopodia and start to spread between the microspheres. The differences between these two substrates rely on the fact that on the LD-M film, cells still have film to adhere to and seem to be flattened when compared to the HD-M film, where cells find themselves on a niche full of microspheres. The tension provided by the LD-M and the flat film on the cells can be larger and cells are able to spread more on these substrates, making them suitable for osteogenic differentiation.

Complementary to these results, the MFC technique was applied to observe how the surface markers evolved over time and on different substrates. It was previously shown that MFC can identify cell differentiation into distinct cell lineages [25]. All cells cultured on the produced biomaterials were analyzed compared to cells of the same passage and at the same day of culture.

The percentages of the expression of CD90, CD105 and CD73 antigens in hMSCs are shown in Table 2. We observed that CD90 expression decreased significantly in hMSCs cultured on all samples, while being more significantly diminished in HD-M, LD-M and film-beta with respect to the control at day 0. The CD105 expression was lower in HD-M samples compared to the control at day 0. In the rest of samples, the decrease of this expression was inferior. HD-M samples showed less expression of CD73 compared with the other samples, which maintained similar levels to control at day 0.

The histograms of hMSCs cultured on TCPS, HD-M, LD-M and β-PVDF films (shown in Figure 4) revealed a large loss of the CD90 surface marker expression when compared to cells cultured at day 0. Furthermore, the HD-M films even presented a subpopulation of cells that lost all of the markers (CD105 and CD73) and had a larger loss of CD90 expression (Figure 4b). The loss of these three hMSC markers together was already reported to be related with their differentiation [22,25]. This leads to the conclusion that both substrate and irregular topography induce the cells to differentiate more than when cultured on flatter surfaces. In Figure 4, it can be observed that there is a larger loss of expression of CD90 and CD105 when compared to CD73 in all the samples, and these differences might be relevant. The loss of CD105 expression is related to multi-lineage differentiation of stem cells [25]. On the other hand, it has been reported that hMSCs lose CD90 expression as cells mature towards osteoblastic-like cells [36]. Low down-regulation of CD73 could be explained by its adenosine production that promotes osteoblast differentiation and by being expressed in mature osteoblasts [37,38]. Thus, its expression may vary, as seen in [25], but not as much as the other positive markers, possibly because more time is still needed for these cells to become totally differentiated, or even because cells could be differentiating into another lineage. Overall, we can conclude that microsphere films, and particularly the HD-M films represent appropriate topography to induce cell differentiation. The diverse topography of biomaterials induces different cell shapes, and these shapes have been shown to indirectly regulate differentiation into the osteoblast phenotype [35]. Therefore, these substrates can be providing the cells with a specific tension that directly stimulates their differentiation, even without addition of supplements or even without reaching confluence, only in the first four days of culture.

To investigate if the osteogenic loss observed with the MFC analysis indicates differentiation towards the osteogenic lineage, the activity of ALP enzyme was examined. This evaluation was performed at day 7, when cells were not yet supplemented with differentiation medium. Furthermore, the results obtained concerning the HD-M films suggest that the adopted cell morphology on these substrates does not favor osteogenic differentiation, and that the loss of mesenchymal stem cell markers observed on the MFC analysis should indicate cell differentiation into another lineage. Also, the LD-M films corroborate this theory, mainly because (as seen in Figure 2) cells still have some gaps of flat film where they can spread further, which is a substrate stiffer than the microspheres (on HD-M films). Stiffer substrates have already been proven to favor osteogenic differentiation [34], which is in agreement with the results for the flat β-phase films. Therefore, the results shown for ALP activity are in agreement with the morphology adopted by the cells and microspheres seem to be inductive of other differentiation pathways.

Finally, after 14 days of osteogenic induction with supplemented medium, osteocalcin localization by immunocytochemistry methods and the quantification of mineralization by alizarin red assay were performed. Regarding the osteocalcin staining (Figure 6), osteogenic differentiation can be confirmed. Also, the microsphere substrates are expressing this major bone protein even more than the glass control. This proves that the support itself has the appropriate features to induce this differentiation state. However, without chemical stimulus, these supports do not directly induce this kind of differentiation. These results are confirmed by the alizarin red results, in which we can confirm that without addition of osteogenic medium, the stiffer or flattened substrates have more calcific deposition. However, it can be seen that, when induced, all of the supports were able to demonstrate calcific deposition, characteristic of osteogenic lineage cells. These results show the capacity of β-PVDF films to enhance hMSC differentiation in early phase (Figure 5) without osteogenic medium. However, based on the alizarin red results, the HD-M film shows higher mineralization (Figure 7). In this way, it is possible to deduce that the β-PVDF films have higher effect at the beginning of differentiation whereas as differentiation progresses, cells grown on HD-M films have an enhanced higher differentiation.

In conclusion, it is possible to say that MFC is a valuable technique for the study of the differentiation of hMSCs. Also, when the topography of the substrate approaches three-dimensionality, the expression of characteristic osteogenic markers of hMSCs decrease. In this way, this study shows the viability of the use of electrosprayed piezoelectric poly(vinylidene fluoride) for tissue engineering applications, which can be used in future in other systems, such as injectable hydrogels.

## 4. Materials and Methods

### 4.1. Processing of Films Adsorbed with Microspheres

PVDF, Solef 1010, was acquired from Solvay and DMF was purchased from Merk. PVDF was dissolved in DMF with a concentration of 7% (*w*/*v*) under magnetic stirring at 60 °C, according the previous work of Correia et al. [26]. An α-phase PVDF film from Measurement Specialties was then subjected to PVDF electrospray, with the previously prepared solution with the same conditions indicated in a former study [26]. Briefly, the polymer solution was placed in a plastic syringe fitted with a steel needle with an inner diameter of 0.25 mm. A syringe pump (NE-1000, Syringepump) fed the polymer solution into the tip at a rate of 2 mL·h^−1^. The distance between the tip of the needle and the collector was 20 cm, the needle being in horizontal position and the collector in vertical position. The experiment was conducted by applying a voltage of 20 kV with a high-voltage power supply (Glassman FC Series 120 W). Two different densities of microparticles adsorbed on film were produced: low (LD-M) and high (HD-M) density. The low density was obtained after 15 min of electrodeposition and the high density after 45 min. “Poled –” β-phase PVDF films (cells cultured on the negatively charged side of the material) from Measurement Specialties were used as control.

The films were cut in 8 mm diameter circles and placed on 48-well non-treated tissue culture polystyrene plates (TCPS, VRW).

### 4.2. Samples Characterization

Electrosprayed samples were coated with a gold layer using a sputter coating (EM MED020, Leica, Wetzlar, Germany) and their morphology was observed by SEM (JSM6300, JEOL, Peabody, MA, USA), with an accelerating voltage of 10 kV. Then, the average diameter of approximately 550 microspheres was measured with the ImageJ Software using the SEM images (https://imagej.nih.gov/ij/).

FTIR was performed at room temperature in a Thermo Nicolet Nexus apparatus in Attenuated Total Reflectance (ATR) mode (GMI, Ramsey, MN, USA). The spectrum was obtained from 4000 to 400 cm^−1^, using 128 scans at a resolution of 8 cm^−1^.

DSC measurements were performed in a PerkinElmer DSC 8000 (PerkinElmer, Villepinte, France) apparatus using a heating rate of 20 °C/min under nitrogen purge.

### 4.3. Materials Sterilization

For sterilization purposes, all the samples were subjected to ultra violet (UV) light overnight and then washed three times for 10 min with Dulbecco's Phosphate-Buffered Saline (DPBS) (Thermo Fisher, Waltham, MA, USA).

### 4.4. Fibronectin Adsorption

Fibronectin (FN) from human plasma (Sigma-Aldrich, St. Louis, MO, USA) was adsorbed onto the PVDF samples. The biomaterials were immersed in a FN solution of 20 μg/mL for 1 h under constant shaking. After protein adsorption, the samples were rinsed in saline solution to eliminate the non-adsorbed protein.

### 4.5. Extraction of Human Mesenchymal Stem Cells

Bone marrow (BM) from patients without hematological malignancies and normal cytomorphological study was collected at the Hematology Department of the Hospital Universitario y Politécnico La Fe of Valencia. This procedure was performed according to established protocols after informed approval of the Local Ethics Committee of the Hospital. The isolation of mononuclear cells (MNC’s) of BM samples was performed by ficoll density gradient centrifugation. Briefly, the BM samples were diluted in Dulbecco’s Modified Eagle’s Medium (DMEM, Thermo Fisher, Waltham, MA, USA) in a proportion 1:2. After, 3 mL of Histopaque^®^-1077 (Sigma-Aldrich) were added to the sample and the mixture was centrifuged at 1000× *g* for 25 min at RT. MNCs at the interphase were collected and washed twice in DMEM at 400× *g* for 10 min. Finally, the MNCs were diluted on DMEM with 10% fetal bovine serum (FBS, Biowest, Labclinic, Nuaillé, France) and counted. Cells were then seeded on T25 cm^2^ flasks (Becton Dickinson, San Jose, CA, USA) with DMEM culture medium composed with 10% FBS, 100 U/mL penicillin-streptomycin (P/S, Invitrogen) and 2.5 mg/L amphotericin B (Sigma Aldrich, St. Louis, MO, USA) at 37 °C in a 95% humidified air containing 5% CO_2_.

After 48 h, the medium was changed and non-adherent cells were discarded. The isolation of hMSCs from the MNCs relies on their ability to adhere on plastic between 24–48 h [39]. The medium changes were performed every 4 days.

### 4.6. Primary Cell Culture and Cell Characterization by Flow Cytometry

When 90% of confluence was reached, cells were trypsinized (trypsin-EDTA 0.25%, Thermo Fisher, Waltham, MA, USA). Then it was centrifuged at 400× *g* for 10 min. After discarding the supernatant, the cells were diluted in low-glucose DMEM supplemented with 10% of FBS. Cells were counted and cultured at a density of 100,000 cells/flask.

The mesenchymal origin of the cells was confirmed by their adherence to tissue culture polystyrene (TCPS) and by surface markers expression using MFC. Freshly obtained and expanded hMSCs (day 0) were characterized by MFC in a FACSCanto-II (Becton Dickinson, San Jose, CA, USA). For this purpose, FcR Blocking Reagent (Miltenyi Biotec, Auburn, CA, USA) was added to the cells in order to block unspecific binding and hMSCs were stained using monoclonal antibodies (MoAbs) fluorochrome conjugated (30 min, 2–8 °C in dark) (Table 1).

A minimum of 50,000 cells were acquired and data were subsequently analyzed using FACSDiva software (Becton Dickinson, San Jose, CA, USA).

### 4.7. Cell Culture

The isolated hMSCs were maintained and expanded in maintenance medium (DMEM containing 1 g/L glucose supplemented with 0.5% amphotericin B, 1% P/S and 10% FBS) at 37 °C in a 95% humidified air containing 5% CO_2_. The medium was changed every 3 days.

The experiments were performed at passages 4–6. A density of 1 × 10^4^ cells/cm^2^ was seeded onto each one of the films (“poled –” β-PVDF films and PVDF films with high and low density of microspheres). Cells were kept under expansion medium until confluence was reached.

The cells that were not used immediately in experiments were placed in cryovials (Thermo Scientific, Waltham, MA, USA) and frozen in liquid nitrogen with FBS supplemented with 10% dimethyl sulfoxide (DMSO) after trypsinization and centrifugation at 400× *g* for 5 min.

Additionally, a differentiation culture medium (osteogenic medium) was added after the hMSCs reached 100% confluence on the biomaterials. The osteogenic medium was composed of DMEM medium containing 1 g/L glucose supplemented with 0.5% amphotericin B, 1% P/S, 10% FBS, 8 mM of β-Glycerophosphate disodium salt hydrate (Sigma Aldrich), 10 nM of dexamethasone-water soluble (Sigma Aldrich, St. Louis, MO, USA) and 50 μg/L of l-Ascorbic acid 2-phosphate sesquimagnesium salt hydrate (Sigma Aldrich, St. Louis, MO, USA). The cell culture medium was replaced every 3 days during 14 days.

### 4.8. Cell Viability

For quantification of viable cells in proliferation, after 4 days of cell seeding on the supports, MTS assay was carried out. To do so, cells were incubated with a 5:1 proportion of MTS (Promega, Madison, WI, USA) to DMEM without phenol red (Thermo Fisher, Waltham, MA, USA) for 3 h at 37 °C in the dark. Then, the supernatant was used to determine the absorbance at 490 nm. For this study, the solution of MTS + DMEM without phenol red was used as reference (blank) and the supernatant of cells cultured in 12 mm glass coverslips were considered to be the positive control.

All the quantitative results will be presented as mean ± standard deviation (SD) of triplicate samples. Statistical differences were determined by ANOVA using Tukey test for the evaluation of different groups (Graphpad Prism 5.0, GraphPad Software). *p* values < 0.05 were considered to be statistically significant.

### 4.9. Cell Morphology

At the fourth day of culture, the morphology of the cells on the different produced PVDF supports was analyzed. First, the samples were fixed with formalin (Sigma Aldrich) at 4 °C for 1 h. The samples were then washed in phosphate buffer (PB) (ThermoFisher, Waltham, MA, USA) before incubation with 1% osmium tetraoxide (Aname) in PB for 45 min in the dark. Then, the biomaterials were again washed to assure total removal of osmium tetraoxide, before being dehydrated through a graded series of alcohol (50%, 60%, 70%, 80%, 96% and 100%) and submitted to critical-point drying (E3000, Polaron, Quorum Technologies, East Sussex, UK). The dried samples were coated with a gold layer using a sputter coating (EM MED020, Leica, Wetzlar, Germany) and their morphology was observed by SEM (JSM6300, JEOL) with an accelerating voltage of 10 kV.

### 4.10. Assessment of Osteogenic Differentiation

#### 4.10.1. Flow Cytometry Study

To analyze the hMSC differentiation, aliquots of cells cultured onto the biomaterials were studied by MFC at day 4 (when approximately 90% of confluence was reached). At this time point, cells do not have osteogenic supplements nor mechanical stress or cell-cell interactions. Therefore, the differentiation produced on cells was due to the PVDF nature and topography.

In order to have a suitable number of cells to perform MFC analysis, biomaterials were cut to a diameter of 3.4 cm and placed in the 6-well non-treated TCPS. Cells were cultured with the same density mentioned previously and, at the day of analysis, they were treated with 1 mL of trypsin for 5 min. Each one of the biomaterial-cultured cells were divided in three aliquots and stained with the antibodies combinations according to Table 3. The following protocol was the same as described above for cells before seeding (day 0).

#### 4.10.2. Study of Alkaline Phosphatase Activity

After 7 days of cell culture (in expansion medium), the cells were lysed and collected. The level of ALP present in the cells was analyzed using a SensoLyte^®^ pNPP Alkaline Phosphatase Assay Kit (ANASPEC) and the total protein of the lysate on each sample was determined using a Micro BCA™ Protein Assay Kit (Thermo Scientific, Waltham, MA, USA). The alkaline phosphatase was expressed as per microgram of total protein for each sample.

#### 4.10.3. Osteocalcin Immunocytochemistry

After 14 days of culture in osteogenic differentiation medium, the content of bone-specific OC was measured by immunocytochemistry methods. First, the cells were washed in DPBS and fixed with formalin as described before. After, hMSCs were washed 3 times with DPBS++ (+calcium, +magnesium, Sigma Aldrich, St. Louis, MO, USA ) and permeabilized with 0.5% Triton X-100 (Sigma Aldrich, St. Louis, MO, USA) in DPBS during 5 min at RT. After washing the samples with DBPS++, a protein solution with 5% of bovine serum albumin (BSA, Sigma Aldrich, St. Louis, MO, USA) and 0.1% Triton X-100 in DPBS was added. After 30 min at 37 °C, the solution was removed and the samples were incubated with anti-osteocalcin antibody (Abcam, Cambridge, UK) at a 1:200 dilution in a solution of 5% BSA and 0.1% Triton X-100 in DPBS for 1 h at 37 °C. Then, the primary antibody was removed and the samples were washed with 0.1% Triton X-100 in DPBS++. At this point, the samples were incubated with the secondary goat antibody anti-rabbit Alexa 488^®^ (Invitrogen, Waltham, MA, USA), at a 1:200 dilution in the previously termed BSA solution for 1 h at 37 °C. Finally, the solution was removed and the samples were once again washed in 0.1% Triton X-100 in DPBS++ before being mounted in a microscope slide with aqueous mounting medium containing DAPI (Vector Laboratories, Peterborough, UK). For this study, cells cultured in 12 mm glass coverslips were used as a reference. Cells relative content of OC was studied using a confocal microscope (DMi8, Leica, Wetzlar, Germany) and ImageJ, Photoshop and Leica Application Suite X software were used for treatment and analysis of the obtained images.

#### 4.10.4. Quantitative Analysis of Alizarin Red Staining

A quantitative analysis of ARS (Sigma-Aldrich, St. Louis, MO, USA) was performed after 14 days of the addition of differentiation medium (expansion, differentiation). For quantification of mineralization, the protocol described in Gregory et al. was followed [40]. This assay is based on alizarin red S staining of the mineral followed by the extraction with 10% acetic acid (Panreac). The acidified ARS is then neutralized by the addition of ammonium hydroxide (Sigma Aldrich, St. Louis, MO, USA). Also, alizarin red standards were prepared with known solution concentrations for the standard curve elaboration. Finally, 50 μL of the sample/standard was added to an opaque-walled, transparent bottom 96-well plate and the absorbance was spectrophotometrically measured at 405 nm. Quantitative analysis was calculated from the standard curve.

### 4.11. Statistical Analysis

All quantitative data were analyzed using GraphPad Prism (v6.00, La Jolla, CA, USA). The results were analyzed statistically using the one way ANOVA test for the cell viability and ALP assays and two-way ANOVA test for the alizarin red assays followed by Tukey’s test. Differences were considered to be significant when *p* < 0.05.

## 5. Conclusions

MFC has proven to be a powerful and valuable technique for the study of hMSC differentiation in cells cultured on biomaterials, giving a quantitative reliable analysis of hMSC-associated markers and the loss, gain or maintenance of marker expression. It has been shown that the decrease of the expression of characteristic markers of multipotentiality in hMSCs is more apparent when the topography of the substrate approaches three-dimensionality. Since these cells lose their specific markers, we can conclude that the supports are naturally inducing cells to differentiate into other lineages. It has been shown that electrosprayed piezoelectric poly(vinylidene fluoride) are valuable for tissue engineering purposes, as hMSCs can proliferate, be viable and undergo osteogenic differentiation when chemically stimulated.

## Figures and Tables

**Table 1:** Percentage of expression of unstained and stained hMSCs surface proteins and number of events analyzed.

hMSCs	CD90 (%)	CD105 (%)	CD73 (%)	HLADR (%)	CD19 (%)	CD34 (%)	CD45 (%)	CD14 (%)	Events
Unstained	0.1	0.1	0.2	0.1	0.1	0.1	0.2	0.1	11,637
Stained	77.7	98.6	99.7	2.4	0.2	0.2	0.1	0.1	12,217

**Table 2:** Percentage of expression of the hMSC specific markers cultured on different biomaterials (TCPS, HD-M, LD-M and film-beta) compared with control at day 0 and number of events analyzed.

**hMSCs**	**CD90 (%)**		**CD105 (%)**		**CD73 (%)**		**Events**
	**Sample**	**Control**	**Sample**	**Control**	**Sample**	**Control**	
TCPS	35.4	77.7	87.3	98.6	99.4	99.7	46,873
High Density	0.8		51.2		79.0		16,893
Low Density	3.9		81.1		97.9		7,156
Film beta	2.7		82.0		99.1		27,355

**Table 3:** MoAbs fluorochrome conjugated employed for surface staining to characterize hMSCs: fluorescein isothiocyanate (FITC); phycoerythrin (PE); peridinin chlorophyll protein-cyanine5.5 (PerCP-Cy5.5); allophycocyanin (APC).

Tube	FITC	PE	PerCP-Cy5.5	APC
1	CD90 ^1^	CD105 ^1^	HLA-DR ^2^	CD73 ^1^
2	CD19 ^2^	CD34 ^2^	CD45 ^2^	CD14 ^2^
Unstained	NA	NA	NA	NA

^1^ Miltenyi Biotec; ^2^ Becton Dickinson; NA: no antibody.

## Supplementary Materials

Supplementary materials can be found at www.mdpi.com/1422-0067/18/11/2391/s1.

## Abbreviations

ALP	Alkaline phosphatase
APC	Allophycocyanin
ARS	Alizarin red staining
ATR	Attenuated Total Reflectance
BM	Bone marrow
DMEM	Dulbecco’s Modified Eagle’s Medium
DMF	*N*,*N*-dimethylformamide
DPBS	Dulbecco’s Phosphate-Buffered Saline
DSC	Differential scanning calorimetry
FBS	Fetal bovine serum
FITC	Fluorescein isothiocyanate
FN	Fibronectin
FTIR	Fourier transformed infrared
HD-M	High density of microparticles
HMSCs	Human mesenchymal stem cells
LD-M	Low density of microparticles
MNC's	Mononuclear cells
MTS	3-(4,5-dimethylthiazol-2-yl)-5-(3-carboxymethoxyphenyl)-2-(4-sulfophenyl)-2H-tetrazolium
P/S	Penicillin-streptomycin
PE	Phycoerythrin
PerCP-cY5.5	Peridinin chlorophyll protein-cyanine5.5
PVDF	Poly(vinylidene fluoride)
RT-PCR	Real-time polymerase chain reaction
SEM	Scanning electron microscope
TCPS	Tissue culture polystyrene plates
