# Supplementary material for: Human Mesenchymal Stem Cells Growth and Osteogenic Differentiation on Piezoelectric Poly(vinylidene fluoride) Microsphere Substrates
Source: Int J Mol Sci. 2017 Nov 11;18(11):2391. doi: 10.3390/ijms18112391 (PMC5713360; doi:10.3390/ijms18112391)
Supplement: Supplementary file 1 [file ijms-18-02391-s001.pdf]

Supplementary Materials: Human mesenchymal stem cells growth and differentiation on piezoelectric poly(vinylidene fluoride) microsphere substrates

R. Sobreiro Almeida, M. N. Tamaño-Machiavello, E. O. Carvalho, L. Cordón, S. Doria, L. Senent, D. M. Correia, C. Ribeiro, S. Lanceros-Méndez, R. S. i Serra, J. L. Gomez Ribelles, A. Sempere

Figure S1 shows representative SEM images of the PVDF microparticles electrosprayed on a PVDF film at low or at high density (Figure S1a and S1b) and the corresponding size distribution (Figure S1c).

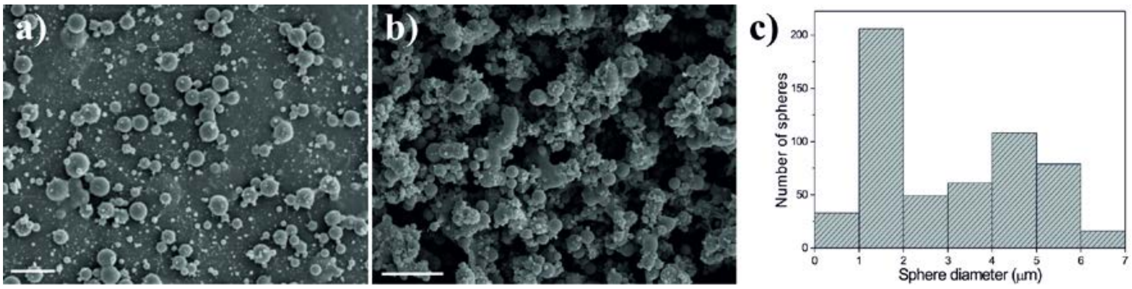

Figure S1 - Morphology of PVDF microspheres electrosprayed on PVDF films at a) low density and b) high density. The scale bar is 10 µm for both samples. c) Microspheres size distribution obtained for the described electrospray conditions.
